# A simple, high-throughput modeling approach reveals insights into the mechanism of gametophytic self-incompatibility

**DOI:** 10.1038/srep34732

**Published:** 2016-10-10

**Authors:** Jahanshah Ashkani, D. J. G. Rees

**Affiliations:** 1 Biotechnology Department, University of the Western Cape, Robert Sobokwe Road, Bellville, 7535, South Africa; 2 Agricultural Research Council, Biotechnology Platform, Private Bag X5, Onderstepoort, 0110, South Africa

## Abstract

Specificity in the GSI response results from the *S*-haplotype-specific molecular interaction of *S*-locus F-box (SLF/SFB) and SRNase proteins in the self-incompatibility locus (*S*-locus). The answer to the question of how these two components of the *S*-locus (SRNase and SLF/SFB) interact has been gathered from several models. Since there is not enough evidence as to which one is the definitive model, none of them can be ruled out. Despite the identification of interacting protein elements, the mechanism by which SLF/SFB and SRNase interact to differently trigger the self-incompatibility among families and subfamilies remain uncertain. The high-throughput modeling approach demonstrates structural visions into the possible existence of a Collaborative Non-Self Recognition model in apple. These findings postulate several prospects for future investigation providing useful information to guide the implementation of breeding strategies.

Gametophytic self-incompatibility (GSI) plays a key role in the genetic diversity within plants while changing patterns of lineage diversification in clades that employ these mechanisms^1^,^2^,^3^,^4^. GSI involves the participation of both factors from the pollen and pistil^5^, which play an important role in pollen recognition and rejection^6^. One of the important features of the GSI is the existence of an extracellular ribonuclease in the pistil, the SRNase (the female determinant of SI), and its involvement in the rejection of incompatible pollen cells^7^. The *Rosaceae* SRNase contains a single hypervariable region (RHV) that is the most hydrophilic part of the SRNase, suggesting that it could be the prime determinant of the SRNase specificity^8^. The high level of allelic polymorphism of the SLF/SFB gene, along with its pollen-specific expression and close physical distance to the SRNase gene in the *S*-locus supports the hypothesis that SLF/SFB is the male determinant of GSI^9^,^10^. Despite the identification of F-box genes as pollen-*S* candidates the mechanism by which SLF/SFB and SRNase interact to trigger the self-incompatibility reaction remains uncertain. Several studies have suggested divergence of pollen-*S* function among different taxa with some having a self-recognition system with a single factor while others represent a non-self recognition system with multiple factors^11^,^12^,^13^,^14^,^15^,^16^,^17^,^18^,^19^. The increase in the number of SLF genes implies that a larger repertoire of SRNases can be targeted, thus allowing more potential coupling partners, while the expansion in the number of SRNases will have the opposite effect. Accordingly, for a plant species it would be advantageous to have multiple SLF genes as this helps prevent inbreeding and provide diversity in plants.

The growing economic importance of fruit crops and the problems associated with their commercial production has placed the molecular basis of self-incompatibility under intense attention. This has led to the rise of many hypotheses about how the pistil *S*-ribonuclease (SRNase) and pollen-expressed *S*-haplotype-specific F-box (SFB) interact. Kubo and colleagues (2010) proposed a Collaborative Non-Self Recognition model for the interaction of SLF variants (brothers) with different SRNases. This model assumes that the pollen *S*-locus encodes several SLF proteins and different SLF variants can recognize a subset of SRNases^20^. It is therefore of great interest to evaluate and study this model in various species, as it provides clues about the mechanism of GSI employed and further assists in formulating future studies, especially those focused on apple due to its economic significance in worldwide agriculture. It is notable that successful breeding programs rely on the availability of diverse germplasm, which poses the need to develop and introduce new cultivars. Additionally, the GSI mechanism represents an obstacle for the breeding of apple by restricting crosses between apple cultivars. As such the knowledge of GSI as to how the SLF/SFB and SRNase proteins interact, can provide useful information to guide the implementation of breeding strategies.

To address this issue a model of interaction between *S*-locus genes is introduced by assessing the Collaborative Non-Self Recognition model in apple. This model aims at describing the specificities of SLF/SFB and their brothers (SFBBs) in recognizing a large repertoire of SRNases, using molecular modeling and docking techniques.

## Results and Discussion

**Figure 1:**
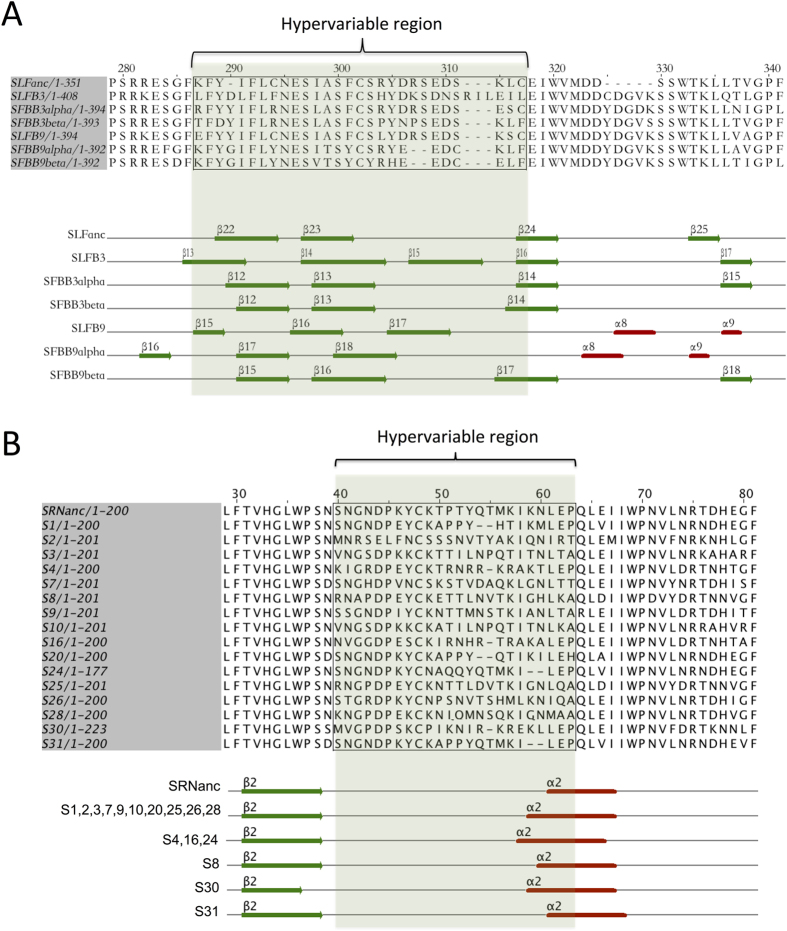
Graphical representations of the location of hypervariable region (HV) on the alignment and secondary structures of *Malus* (**A**) SLF/SFBs and (**B**) SRNases. Highlighted regions on the primary and secondary structure of SLF/SFBs and SRNases are shown the location of HV region while ‘S’ referrers to SRNase. Ancestor sequences of SLF/SFBs and SRNases (*i.e*. SLFanc and SRNanc) were reconstructed using the Bayesian method as described by Hall^35^ as described by Ashkani and Rees^23^. HV regions containing 27 amino acids (from Lys-247 to Cys-273, on loop 19 and a section of β16-sheet) and 24 amino acids (from Ser-40 to Pro-63, on loop 2 and a section of α2-helix) were identified for SLF/SFBs and SRNases based on the ancestor amino acid sequences, respectively.

With the aim of inspecting the Collaborative Non-Self Recognition model in apple, *Malus* × *domestica* (Borkh.), the tertiary structure of known SRNases, along with SLFB3 and SLFB9, as well as their relevant brothers namely SFBB3α, SFBB3β and SFBB9α and SFBB9β, were predicted using the I-TASSER suite^21^ (Table S1-S7 and Fig. S1-S8). The folding topologies of the modeled SRNases structures were examined using the Dali server, which were found to be very similar to the topologies of *Momordica charantia* ribonuclease MC and MC1 (Table S4). Hence, it is concluded that these predicted structures have good homology with the experimentally solved structures of the RNase T2 family enzymes. These models can therefore be used confidently in a docking study to further assess the Collaborative Non-Self Recognition model in apple. With respect to SLF/SFBs and their relative brothers (SFBBs), the folding topologies of the modeled structures were found to be commonly very similar to the topologies of F-BOX/WD-repeat proteins (Table S3). Therefore, the result of Dali server clearly shows that the template and modeled structures could be part of the same protein family. Previous protein binding studies have shown that SLF interacts with the hypervariable (HV) region of S-RNase^22^, accordingly in this analysis only the residues within the predicted hypervariable region (Fig. 1) as identified previously by Ashkani and Rees^23^ were used as the interacting interface.

ZDOCK^24^,^25^,^26^ was used to predict SLF and SRNase interactions in *S*-locus while the docking results were narrowed down to those predicted complexes that have shown a significant interaction based on the Wilcoxon rank-sum test (p-value < 0.0001) comparing binding energies (ZRANK scores). The null hypothesis for this analysis was based on the finding of Hua and Kao (2006) who suggested that the non-self interactions are much stronger than the self-interactions^22^. The accuracy of the predicted complexes by docking was further assessed using the root mean square deviation (RMSD) between the atoms of the predicted complex and the native or near-native complex, the model with the best binding energy (Figs S9 and S10).

**Figure 2:**
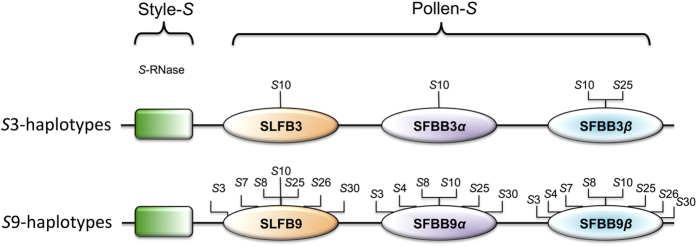
Graphical representation of the introduced model of interaction between *Malus* S-locus proteins. The figure was generated based on the results of Wilcoxon rank-sum test^33^. SLFB3 and SLFB9, and their brothers (SFBB3α, SFBB3β, SFBB9α, SFBB9β) are all predicted to interact with S10-RNase. In addition SFBB3β interact with S25 specifically. In terms of SLFB9 and its brothers (SFBB9α, SFBB9β), they all interact with S3-, S8-, S25- and S30-RNase with different binding affinities. However, SLFB9 and SFBB9β interact with S7- and S26-RNase, and SFBB9α and SFBB9β interact with S4-RNase. ‘S’ referrers to SRNase.

The results of the Wilcoxon rank-sum test (Table 1) shows that SFBB3β from the S3-haplotype interacts more strongly with S10- and S25-RNase compared to the self S3-RNase while SLFB3 and SFBB3α are only strongly interacting with S10-RNase. With regards to SLFB9 and its brothers (SFBB9α and SFBB9β), they all commonly interact with S3-, S8-, S10-, S25- and S30-RNase. In addition, SLFB9 and SFBB9β commonly interact with S7- and S26-RNase while SFBB9α and SFBB9β commonly interact with S4-RNase (Fig. 2).

Therefore, it is suggested that the self/non-self recognition system in apple is controlled by multiple factors where SLF/SFBs interact with non-self SRNases with different binding specificities and thus proteins originally identified as SLF-like (SLF/SFB brothers), may indeed be true SLF/SFB proteins in carrying out the process of self/non-self recognition. Consequently, the presence of extra subsets of SLF/SFB proteins in the *S*-locus, which can interact with certain subset of SRNases is expected while additional SLF proteins in *S*-locus have yet to be characterised in *Malus*. In line with this hypothesis, Minamikawa and colleagues^18^, and Okada and co-workers^27^ reported additional SFBB-like genes/alleles in apple, and it is likely that more SFBB genes remain to be identified. However, recently multiple male factors in pear (*Pyrus pyrifolia*) have also been identified^28^. In addition, common binding of a specific subset of SRNases to another subset of SLF/SFBs, are consistent with the scenario that a large repertoire of non-self SRNases are targeted and detoxified by multiple SLF/SFBs each of which recognizes a sub-fraction of SRNases^28^,^29^. These findings are in line with the Collaborative Non-Self Recognition model that the loss-of-function of one of multiple SLF/SFBs leads to a limited effect on the GSI phenotype.

Though our findings pertaining to the computational prediction of SLF/SFB and SRNase mechanism of interaction hold great value for future breeding studies, its further validation through direct protein-protein interaction studies is needed.

## Conclusion

It is known that the specificity of the self-incompatibility response results from *S*-haplotype-specific molecular interactions of SLF/SFBs and SRNases. As such understanding the recognition mechanism of GSI requires detailed structure-function analysis of the *S*-locus proteins. Such structure-function studies require an experimental system that allows efficient *in vivo* functional analysis of large numbers of SLF and SRNase variants generated *in vitro* by site-directed mutagenesis or domain swapping between proteins that determine different GSI specificities. However, due to the experimental limitations associated with such studies, the computational approach undertaken here represents a powerful alternative. Hence, these findings provide the basis for studying the appearance of multiple subsets of SLF/SFBs and the biochemical basis that allows a particular subset of SLF/SFBs to recognize certain subset of SRNases but not others.

## Methods

The I-TASSER suite^21^ was used to predict the tertiary structures of SLFB3, SFBB3α, SFBB3β, SLFB9, SFBB9α, SFBB9β and SRNase haplotypes (*i.e*. S1-S4, S7-S10, S16, S20, S24-S26, S28, S30, S31) (Table S1). The quality of the final refined models was subjected to a series of tests for their internal consistency and reliability. Backbone conformation and overall model quality were evaluated by PROCHECK^30^ in VADAR v1.8^31^ and ProSa-web^32^, respectively. Furthermore, the folding topologies of the modeled structures were examined using the Dali server (http://ekhidna.biocenter.helsinki.fi/dali_server). The ZDOCK program was used to evaluate the interactions between currently known *Malus x domestica* (Borkh.) SLF/SFBs and SRNases. The PDB file of the modeled structures of SLF/SFBs and SRNases were used as inputs to ZDOCK as receptors and ligands respectively. For docking purpose, the number of top poses for SLF/SFB-SRNase complex was set to 2 000 with the root mean square division (RMSD) cut off the value of 10 while only the hypervariable regions identified by Ashkani and Rees^23^ was provided to ZDOCK as an interface. Furthermore, ZRANK was used to re-rank the ZDOCK scores while the binding energies from ZRANK were analysed using Wilcoxon rank-sum test^33^, as implemented in R^34^. The RMSD values were calculated with ProFit v3.1 (Martin, unpublished, http://www.bioinf.org.uk/software/profit). Assuming that a pose with the lowest binding energy is the nearest native, the structure with the top ZRANK from the previous analysis was provided to ProFit as the reference structure in PDB format. All the other structures (from 2 000 poses) were provided as mobile structures. The reference structure remains fixed while the mobile structures are fitted on to it. Finally, to determine the accuracy of the scoring function for docking models that were shown to be significant using Wilcoxon rank-sum test, the ZRANK scores were plotted against ligand RMSDs, L-RMSDs. Plotting the ZRANK scores against LRMSDs shows the distribution of the poses in the form of an energy funnel, which is suggestive of the accuracy of the scoring function, whereby most near-native complexes have lower binding energies and low L-RMSDs.

## Additional Information

**How to cite this article**: Ashkani, J. and Rees, D. J. G. A simple, high-throughput modeling approach reveals insights into the mechanism of gametophytic self-incompatibility. *Sci. Rep*. **6**, 34732; doi: 10.1038/srep34732 (2016).

## Supplementary Material

Supplementary Information

## Figures and Tables

**Table 1:** The predicted binding energies for the interacted SLF/SFBs and SRNases.

SRNase	SLFB3	SFBB3α	SFBB3β	SLFB9	SFBB9α	SFBB9β
S1	−35.61	−29.55	−31.75	−25.85	−32.30	−35.17
S2	−28.58	−17.01	−24.68	−21.49	−27.29	−30.87
S3	**(−49.01)**	**(−53.49)**	**(−51.55)**	−38.77*	−44.54*	−54.07*
S4	−41.83	−38.62	−43.12	−31.83	−39.08*	−42.70*
S7	−35.79	−41.07	−37.81	−36.33*	−36.25	−43.32*
S8	−42.55	−43.74	−48.75	−36.73*	−43.69*	−47.39*
S9	−35.90	−34.37	−41.61	**(−30.65)**	**(−34.37)**	**(−41.61)**
S10	−56.37*	−61.77*	−59.89*	−47.87*	−48.72*	−60.64*
S16	−35.09	−29.84	−31.54	−25.69	−33.20	−35.39
S20	−37.17	−32.61	−32.68	−28.55	−35.22	−39.99
S24	−37.88	−33.88	−36.30	−28.21	−32.86	−40.57
S25	−46.93	−46.41	−53.37*	−35.65*	−45.87*	−51.96*
S26	−42.65	−42.88	−43.17	−36.17*	−35.61	−47.35*
S28	−32.52	−30.22	−29.92	−25.15	−28.67	−35.65
S30	−38.69	−35.47	−38.55	−32.29*	−38.31*	−43.45*
S31	−36.95	−34.28	−37.40	−28.21	−34.42	−40.39

The non-self interactions were statistically assessed using Wilcoxon rank-sum test^33^. The null hypothesis assumes that no interaction exists between SLF/SFBs and non-self SRNases if binding energy in the non-self interaction is more than or equal to that of self interactions.

Note: *Significant (p < 0.0001), bold values in the parenthesis show the binding energies for the self-interactions.

## References

[b1] Igic B., Lande R. & Kohn J. R. Loss of self-incompatibility and its evolutionary consequences. Int J Plant Sci 169, 93–104 (2008).

[b2] Goldberg E. E. . Species Selection Maintains Self-Incompatibility. Science 330, 493–495 (2010). 20966249 10.1126/science.1194513

[b3] Franklin-Tong N. V. & Franklin F. C. Gametophytic self-incompatibility inhibits pollen tube growth using different mechanisms. Trends Plant Sci 8, 598–605 (2003). 14659709 10.1016/j.tplants.2003.10.008

[b4] Waligorski P. & Szaleniec M. Prediction of white cabbage (Brassica oleracea var. capitata) self-incompatibility based on neural network and discriminant analysis of complex electrophoretic patterns. Comput Biol Chem 34, 115–121 (2010). 20347393 10.1016/j.compbiolchem.2010.03.002

[b5] East E. M. The distribution of self-sterility in flowering plants. Proc Am Philos Soc 82, 449–518 (1940).

[b6] Wheeler M. J., Franklin-Tong V. E. & Franklin F. C. H. The Molecular and genetic basis of pollen-pistil interactions. New Phytol 151, 565–584 (2001). 10.1046/j.0028-646x.2001.00229.x 33853259

[b7] Kao T. H. & Tsukamoto T. The Molecular and genetic bases of S-RNase-based self-incompatibility. Plant Cell 16 Suppl, S72–S83 (2004). 15010517 10.1105/tpc.016154 PMC2643390

[b8] Tsai D. S., Lee H. S., Post L. C., Kreiling K. M. & Kao T. h. Sequence of an S-protein of Lycopersicon peruvianumand comparison with other solanaceous S-proteins. Sex Plant Reprod 5, 256–263 (1992).

[b9] Sijacic P. . Identification of the pollen determinant of S-RNase-mediated self-incompatibility. Nature 429, 302–305 (2004). 15152253 10.1038/nature02523

[b10] Sakai S. & Wakoh H. Initial Invasion of Gametophytic Self-Incompatibility Alleles in the Absence of Tight Linkage between Pollen and Pistil S Alleles. Am Nat 184, 248–257 (2014). 25058284 10.1086/676942

[b11] Takayama S. & Isogai A. Self-incompatibility in plants. Annu Rev Plant Biol 56, 467–489 (2005). 15862104 10.1146/annurev.arplant.56.032604.144249

[b12] Takayama S. . Direct ligand-receptor complex interaction controls Brassica self-incompatibility. Nature 413, 534–538 (2001). 11586363 10.1038/35097104

[b13] Wheeler M. J. . Identification of the pollen self-incompatibility determinant in Papaver rhoeas. Nature 459, 992–995 (2009). 19483678 10.1038/nature08027 PMC2699350

[b14] Eaves D. J. . Self-incompatibility in Papaver: advances in integrating the signalling network. Biochem Soc Trans 42, 370–376 (2014). 24646246 10.1042/BST20130248

[b15] Entani T. . Comparative analysis of the self-incompatibility S-locus region of *Prunus mume*: identification of a pollen-expressed F-box gene with allelic diversity. Genes to Cells 8, 203–213 (2003). 12622718 10.1046/j.1365-2443.2003.00626.x

[b16] Zhou J. . Structural and transcriptional analysis of S-locus F-box genes in Antirrhinum. Sex Plant Reprod 16, 165–177 (2003).

[b17] Sassa H. . S-locus F-box brothers: multiple and pollen-specific F-box genes with S haplotype-specific polymorphisms in apple and Japanese pear. Genetics 175, 1869–1881 (2007). 17237509 10.1534/genetics.106.068858 PMC1855134

[b18] Minamikawa M. . Apple S-locus region represents a large cluster of related, polymorphic and pollen-specific F-box genes. Plant Mol Biol 74, 143–154 (2010). 20628788 10.1007/s11103-010-9662-z

[b19] Minamikawa M. F., Koyano R., Kikuchi S., Koba T. & Sassa H. Identification of SFBB-Containing Canonical and Noncanonical SCF Complexes in Pollen of Apple (Malus × domestica). PLoS One 9, e97642 (2014). 24847858 10.1371/journal.pone.0097642 PMC4029751

[b20] Kubo K.-i. . Collaborative Non-Self Recognition System in S-RNase-Based Self-Incompatibility. Science 330, 796–799, (2010). 21051632 10.1126/science.1195243

[b21] Roy A., Kucukural A. & Zhang Y. I-TASSER: a unified platform for automated protein structure and function prediction. Nat Protoc 5, 725–738 (2010). 20360767 10.1038/nprot.2010.5 PMC2849174

[b22] Hua Z. & Kao T. H. Identification and characterization of components of a putative petunia S-locus F-box-containing E3 ligase complex involved in S-RNase-based self-incompatibility. Plant Cell 18, 2531–2553 (2006). 17028207 10.1105/tpc.106.041061 PMC1626602

[b23] Ashkani J. & Rees D. J. G. A Comprehensive Study of Molecular Evolution at the Self-Incompatibility Locus of Rosaceae. J Mol Evol 82, 128–145 (2016). 26714486 10.1007/s00239-015-9726-4

[b24] Zhang C., Liu S. & Zhou Y. Docking prediction using biological information, ZDOCK sampling technique and clustering guided by the DFIRE statistical energy function. Proteins 60, 314–318 (2005). 15981255 10.1002/prot.20576

[b25] Wiehe K. . ZDOCK and RDOCK performance in CAPRI rounds 3, 4, and 5. Proteins Struct Funct Bioinf 60, 207–213 (2005). 10.1002/prot.20559 15981263

[b26] Chen R., Li L. & Weng Z. ZDOCK: An initial-stage protein-docking algorithm. Proteins Struct Funct Bioinf 52, 80–87 (2003). 10.1002/prot.10389 12784371

[b27] Okada K., Moriya S., Haji T. & Abe K. Isolation and characterization of multiple F-box genes linked to the S9-and S10-RNase in apple (Malus x domestica Borkh.). Plant Reprod 26, 101–111 (2013). 23686223 10.1007/s00497-013-0212-0

[b28] Kakui H. . Sequence divergence and loss-of-function phenotypes of S locus F-box brothers genes are consistent with non-self recognition by multiple pollen determinants in self-incompatibility of Japanese pear (Pyrus pyrifolia). Plant J 68, 1028–1038 (2011). 21851432 10.1111/j.1365-313X.2011.04752.x

[b29] Entani T. . Ubiquitin-proteasome-mediated degradation of S-RNase in a solanaceous cross-compatibility reaction. Plant J 78, 1014–1021 (2014). 24689760 10.1111/tpj.12528

[b30] Laskowski R. A., MacArthur M. W., Moss D. S. & Thornton J. M. PROCHECK: a program to check the stereochemical quality of protein structures. J Appl Crystallogr 26, 283–291 (1993).

[b31] Willard L. . VADAR: a web server for quantitative evaluation of protein structure quality. Nucleic Acids Res 31, 3316–3319 (2003). 12824316 10.1093/nar/gkg565 PMC168972

[b32] Wiederstein M. & Sippl M. J. ProSA-web: interactive web service for the recognition of errors in three-dimensional structures of proteins. Nucleic Acids Res 35, W407–W410 (2007). 17517781 10.1093/nar/gkm290 PMC1933241

[b33] Wilcoxon F. Individual comparisons by ranking methods. Biometr Bull 1, 80–83 (1945).

[b34] R-Core-Team. R: A language and environment for statistical computing. R Foundation for Statistical Computing. R Foundation for Statistical Computing, Vienna, Austria. URL http://www.R-project.org/ (2013).

[b35] Hall B. G. Simple and accurate estimation of ancestral protein sequences. Proc Natl Acad Sci 103, 5431–5436 (2006). 16567642 10.1073/pnas.0508991103 PMC1459372

