# Supplementary material for: A simple, high-throughput modeling approach reveals insights into the mechanism of gametophytic self-incompatibility
Source: Sci Rep. 2016 Oct 10;6:34732. doi: 10.1038/srep34732 (PMC5056379; doi:10.1038/srep34732)
Supplement: Supplementary Information [file srep34732-s1.pdf]

A simple, high-throughput modeling approach reveals insights into the mechanism of gametophytic self-incompatibility

J. Ashkani*[1, 2], D.J.G. Rees[1, 2]

[1]Biotechnology Department, University *of the* Western Cape, Robert Sobokwe Road, Bellville, 7535, South Africa
[2]Agricultural Research Council, Biotechnology Platform, Private Bag X5, Onderstepoort, 0110, South Africa
*Correspondence to: jahanshah.a@gmail.com

Supplementary Materials:
Materials and Methods
Figs. S1 to S10
Tables S1 to S7
References

Materials and Methods

The I-TASSER suite[1] was used to predict the tertiary structures of SLFB3, SFBB3α, SFBB3β, SLFB9, SFBB9α, SFBB9β and SRNase haplotypes (*i.e.* S1-S4, S7-S10, S16, S20, S24-S26, S28, S30, S31) (Supplementary Table 1). This was achieved by running the I-TASSER Perl script (runI-TASSER.pl) which includes the following general steps: *i*) standardizing fasta formatted input sequences to be used by the program *ii*) running PSI-BLAST to identify homologous sequences from NCBI non-redundant database and the PDB database *iii*) running PSIPRED to predict the secondary structures *iv*) executing solve to determine macromolecular structure by scaling data and calculating electron density map and combining the experimental X-ray diffraction data *v*) running the threading programs sequentially an using mkinit.pl to generate restraints, *vii*) performing I-TASSER simulation containing 14 independent runs, *viii*) running the SPICKER clustering program followed by get cscore.pl to get the confidence score, *ix*) finally the EMrefinement.pl is run to generate the full atomic models.

Supplementary Table 2 shows the best templates for modeling the structure of SRNases, whereby a list of corresponding PDB hits along with %Identity, %Coverage, and normalized Z-score values are presented. Among the PDB hits three are common templates for predicting all SRNases, namely *Pyrus pyrifolia* S3-RNase (1iqqA), *Nicotiana glutinosa* ribonuclease NW (1iybA), and *Momordica charantia* ribonuclease MC1 (1bk7A) (Supplementary Figure 1). Furthermore, the percentage sequence identity of the templates in the threading aligned region with the query sequences ranges between 24 − 95%, while the coverage of the threading alignment is between 93 − 100% suggesting that most residues of the query sequences have aligned. Additionally, the normalized Z-score provided by LOMETS threading alignment is in the range of 3.92 − 5.63 (Z-score >1) suggesting a good alignment (Supplementary Table 2). To assess the accuracy of the predicted models the C-scores, TM-scores, RMSD, folding energies, Core Ram (percentage of amino acids in the core region of Ramachandran plot), and ProSa-web Z-score are summarized in Supplementary Table 3. The C-scores for SRNases is between 1.73 − 1.86, while their TM-scores and RMSDs range between 0.96±0.05 − 0.98±0.05 and 1.7±1.5 − 2.0±1.6, respectively. Moreover, the folding energies of the predicted models are between -135.08 and -181.15. In addition, the distribution of the ϕ/ψ angles of most amino acids (96 − 98%) of the modeled structures are in the core and allowed region of the Ramachandran plots (Supplementary Figure 2 and Core Ram values in Supplementary Table 3). To further confirm

the quality of the predicted structures the ProSa-web Z-scores are between -6.11 and -7.16, all of which are within the range of the experimentally predicted structures (Supplementary Figure 3). The folding topologies of the modeled structures were examined using the Dali server, which was found to be very similar to the topologies of *Momordica charantia* ribonuclease MC and MC1, with RMSD value of 2.0 − 2.3 and the identity of 28 − 31% (Supplementary Table 4). Hence, it is concluded that these predicted structures have good homology with the experimentally solved structures of RNase T2 family enzymes. These models can, therefore, be confidently used in the docking study to further assess the Collaborative Non-Self Recognition model in apple, *Malus × domestica* (Borkh.). The final tertiary structures of SRNases were shown in figure S4 and their α+β counts are as follows: *i*) S1- and S2-RNase contain six α-helices and four β-sheets, *ii*) S3-, S8-, and S31-RNase contain seven α-helices and seven β-sheets, *iii*) S4-, S16-, S26-RNase consist of seven α-helices and five β-sheets, *iv*) S7- and S9-RNase contain six α-helices and seven β-sheets, *v*) S10- and S24-RNase consist of seven α-helices and four β-sheets, *vi*) S20-RNase has seven α-helices and six β-sheets, and *vii*) S25-, S28-, and S30-RNase contain six α-helices and five β-sheets.

With respect to SLF/SFBs, Supplementary Table 4 shows the best templates used for modeling the structures and includes a list of PDB hits along with %Identity, %Coverage, and Norm Z-score values. Among the PDB hits used for the prediction four are common, namely *Mus Musculus* Skp1-FbS1 complex (2e31A), *Homo sapiance* Fbw7-Skp1-cyclin E complex (2ovpB), *E. coli* β-propeller protein YghT (2uvkA), *Saccharomyces cerevisiae* Cdc4/Skp1-SCF-I2 (3mksA) (Supplementary Figure 5). The general functional theme of these proteins allows one to elucidate that the predicted structure shares common binding sites with the above-mentioned proteins that allow for their interaction in a similar manner typical of Skp1-Fbox protein. Generally, an F-box protein allows for the formation of SCF (Skp1/Cullin/F-box) E3 ligase complex resulting in the interaction of the F-box motif with the Skp1 protein. Therefore, this information provides further support for the validity of the predicted structures. Furthermore, the percentage sequence identity of the templates in the threading aligned region with the query sequences ranges between 6 − 22% while the coverage of the threading alignment is between 21 − 98% suggesting that many residues of the query sequences have aligned. Additionally, the Norm Z-score provided by LOMETS threading alignment is in the range of 1.06-3.15 (Z-score >1) presenting an acceptable alignment (Supplementary Table 5). To determine the best model from the top five predicted models by the I-TASSER suite the C-score, QMEAN-norm score and folding energies were assessed (Supplementary Table 6). The C-score for the predicted models ranges from -1.34 to -4.29 with the QMEAN-norm score between 0.17 and 0.35 and folding energies between -309.98 and -396.80. According to these results model 1 among the top five models of SFBB3α and SFBB3β, model 2 for SLFB9 and SFBB9β, and model 4 for SLFB3 and SFBB9α were selected as the best models and are highlighted in Supplementary Table 6. The quality of the final refined models was subjected to a series of tests for their internal consistency and reliability. Backbone conformation and overall model quality were evaluated by PROCHECK[2] in VADAR v1.8[3] and ProSa-web[4], respectively. PROCHECK provides a detailed assessment of the stereochemistry of a protein structure as compared with well-defined structures of the same resolution while highlighting regions that may require further investigation[2]. Furthermore, the ProSa-web is used in the refinement and validation of protein structures and provides the quality scores in the context of known protein structures, as well as energy plots that indicate possible problems in the protein structure[4]. The Ramachandran plots represented in Supplementary Figure 6 were generated for the top models from Supplementary Table 6.

According to these figures, the distribution of the φ/ψ angles of most amino acids (90 − 96%) of the modeled structures is in the core and allowed region of the Ramachandran plots. To further support the quality of the predicted models ProSa-web Z-scores are between -4.11 and -6.82, all of which are within the range of the experimentally predicted structures (Supplementary Figure 7). The final tertiary structures of the predicted models were shown in Supplementary Figure 8 and their α+β counts are as follows: *i*) SLFB3 contains 10 α-helices and 23 β-sheets, *ii*) SFBB3α contain eight α-helices and 19 β-sheets, *iii*) SFBB3β consist of 9 α-helices and 17 β-sheets, *iv*) SLFB9 contain eight α-helices and 22 β-sheets, *v*) SFBB9α consist of 9 α-helices and 20 β-sheets, and *vi*) SFBB9β contains 10 α-helices and 21 β-sheets. Furthermore, the folding topologies of the modeled structures were examined using the Dali server, which was found to be generally very similar to the topologies of F-BOX/WD-repeat proteins (Supplementary Table 7), which generally function as platforms of protein-protein interactions and are involved in the various biological process[5].

Since, the hypervariable regions are involved in the control of allele specificity, to identify the location of hypervariable regions on *Malus* SRNases and SLF/SFBs, the previously identified hypervariable regions by Ashkani and Rees (2016) containing 24 amino acids from Ser-40 to Pro-63 and 27 amino acids from Lys-247 to Cys-273, based on ancestor sequence, were mapped on the primary and secondary structures of *Malus* SRNases and SLF/SFBs, respectively. To achieve this, McRate v1[7] was used to determine site-specific evolutionary rates. The evolutionary rate for each site was averaged with that of its neighbors using a sliding window of the size of 11-13 amino acid[6]. The position of the HV regions of SLF/SFBs and SRNases were shown in Figure 1. These results are in line with the findings of Vieira and colleagues (2007) who reported a HV region in the C-terminus of amino acid positions 41-61 for *Malus/Pyrus* SRNase[8]. For SLF/SFBs a HV region was detected in the N-terminus of SLF/SFBs, which is consistent with the results obtained by Tao and colleagues (2010) with the only exception that they described two HV regions (HVa and HVb) in *Prunus* SLF/SFBs [9].

The ZDOCK program was used to evaluate the interactions between currently known *Malus × domestica* (Borkh.) SLF/SFBs and SRNases. The PDB file of the modeled structures of SLF/SFBs and SRNases were used as inputs to ZDOCK as receptors and ligands respectively. For this analysis, the number of top poses for SLF/SFB-SRNase complex was set to 2 000 with the root mean square division (RMSD) cut off the value of 10 while only the hypervariable region was provided to ZDOCK as an interface for docking. Furthermore, ZRANK was used to re-rank the ZDOCK scores. The binding energies from ZRANK were analysed using Wilcoxon rank-sum test[10], as implemented in the R-package [11]. The RMSD values were calculated with ProFitv3.1 (Martin, unpublished, http://www.bioinf.org.uk/software/profit). Assuming that a pose with the lowest binding energy is the nearest native, the structure with the top ZRANK from the previous analysis was provided to ProFit as the reference structure in PDB format. All the other structures (from 2 000 poses) were provided as mobile structures. The reference structure remains fixed while the mobile structures are fitted on to it. Finally, to determine the accuracy of the scoring function for docking models that were shown to be significant using Wilcoxon rank-sum test, the ZRANK scores were plotted against ligand RMSDs, L-RMSDs. Plotting the ZRANK scores against L-RMSDs shows the distribution of the poses in the form of an energy funnel, which is suggestive of the accuracy of the scoring function, whereby most near-native complexes have lower binding energies and low L-RMSDs. The top five poses are highlighted by a circle in each scatter plot and were further superimposed to show the range of positions of the docked ligands (Supplementary Figure 9). The conformational similarities of the

top five poses are clearly observed in Supplementary Figure 10 and it suggests that the top five poses show the probability of binding at a distinct interaction location within the HV region.

Supplementary Figures:

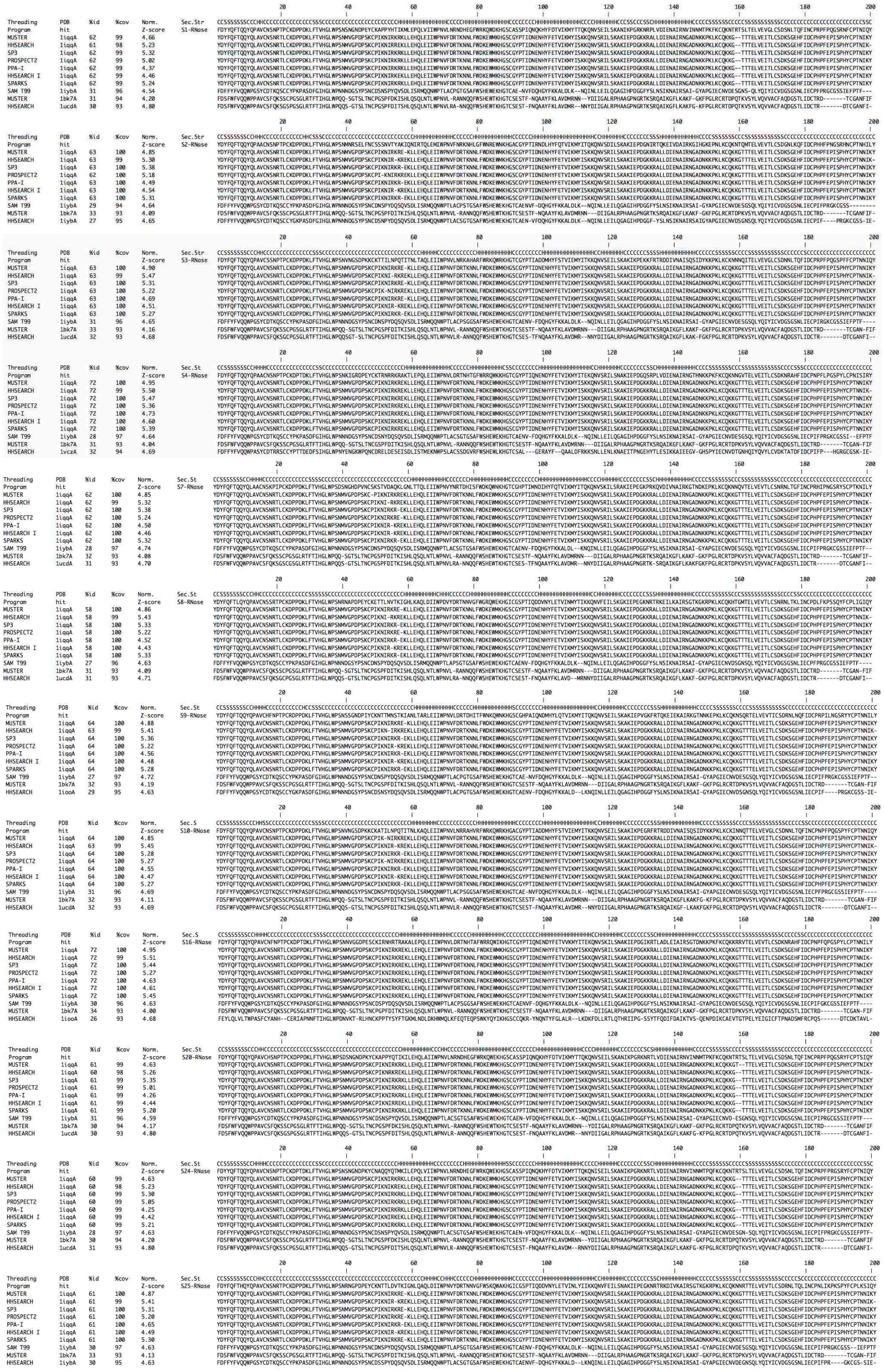

Supplementary Figure 1 | Amino acids threading alignment of the top-ten templates identified by LOMETS threading programs for modeling of SRNases tertiary structures. 1iqqA: *Pyrus pyrifolia*

S3-RNase[12], 1iybA: *Nicotiana glutinosa* ribonuclease NW[13], 1bk7A: *Momordica charantia* ribonuclease[14], 1ucdA: *Momordica charantia* Ribonuclease MC1[15], 1vczA: *Nicotiana glutinosa* ribonuclease NT[16], 1iooA: *Nicotiana alata* S(F11)-RNase[17].

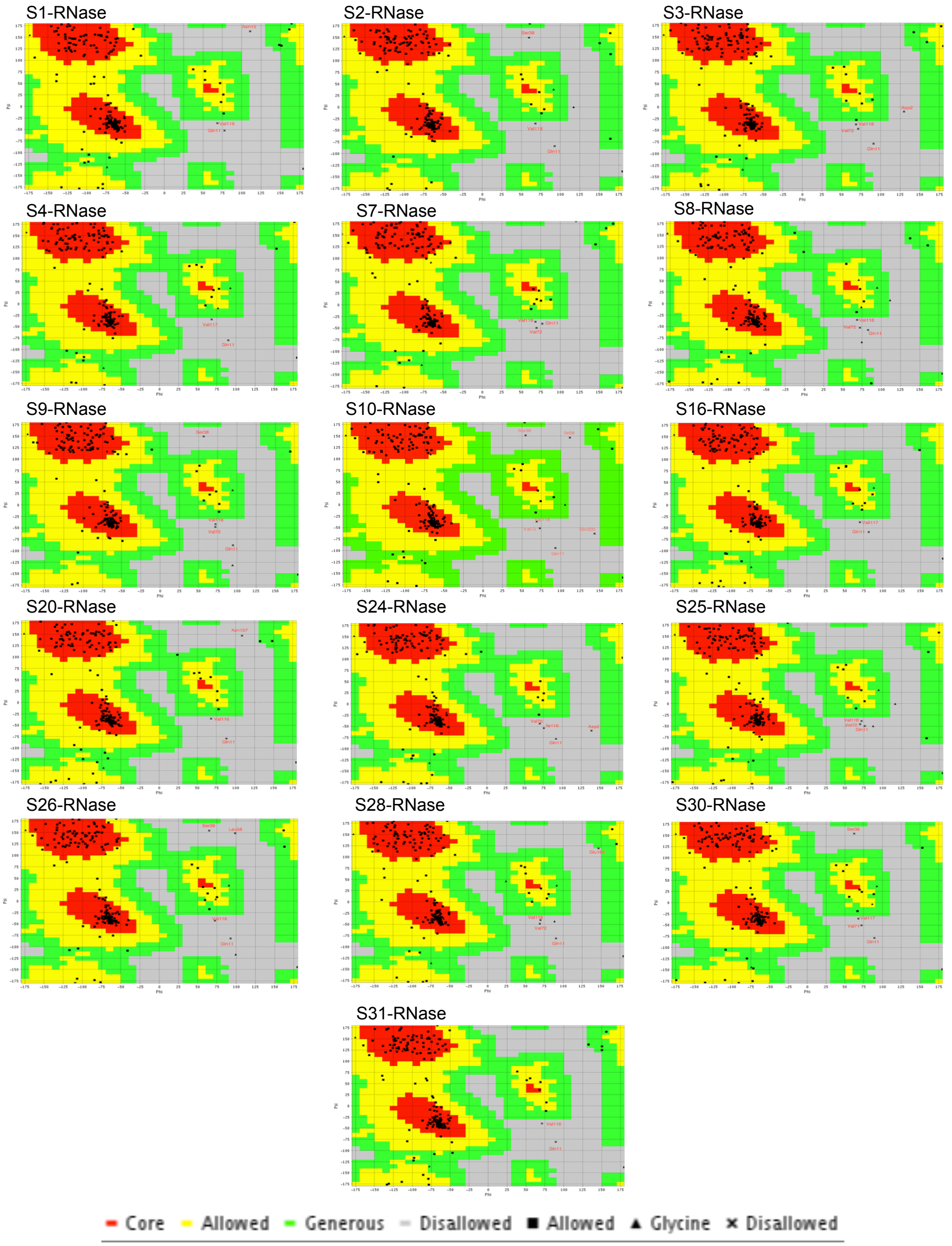

Supplementary Figure 2 | Ramachandran plots of the modeled SRNases. The red, yellow, green, and gray areas refer to core, allowed, general, and disallowed regions of the plot respectively. The plot was generated using PROCHECK[2] in VADAR v1.8[3].

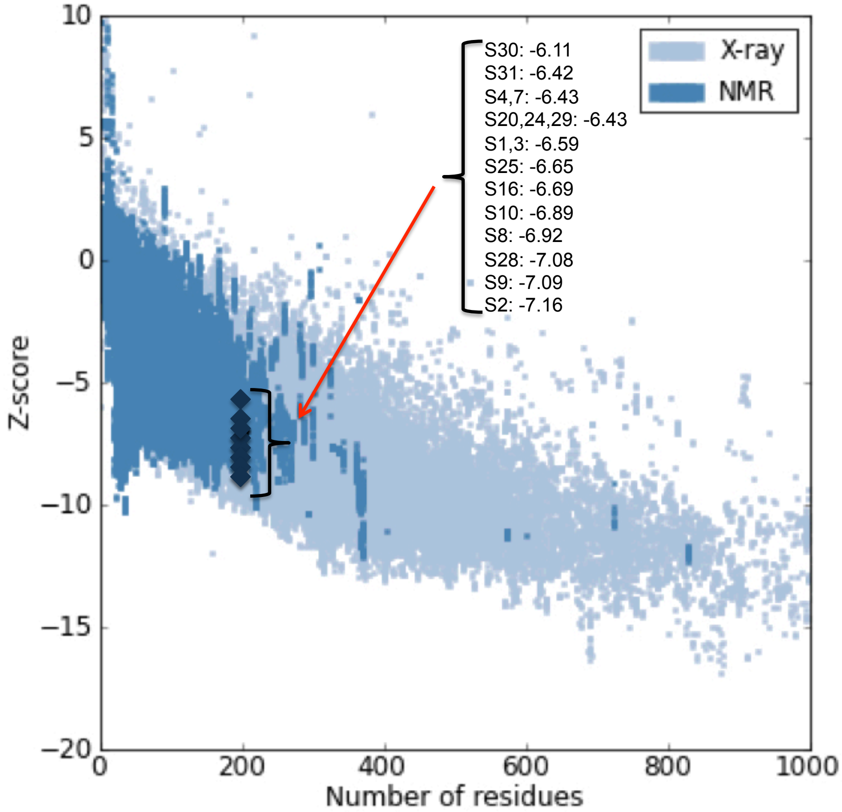

Supplementary Figure 3 | Z-score plot of all experimentally determined proteins. Protein structures from NMR experiments are coloured in dark blue whereas those from X-ray experiment are indicated by light blue. The location of SRNases' z-scores is within the boundaries of experimentally determined structures. The plot was generated using ProSa-web[4].

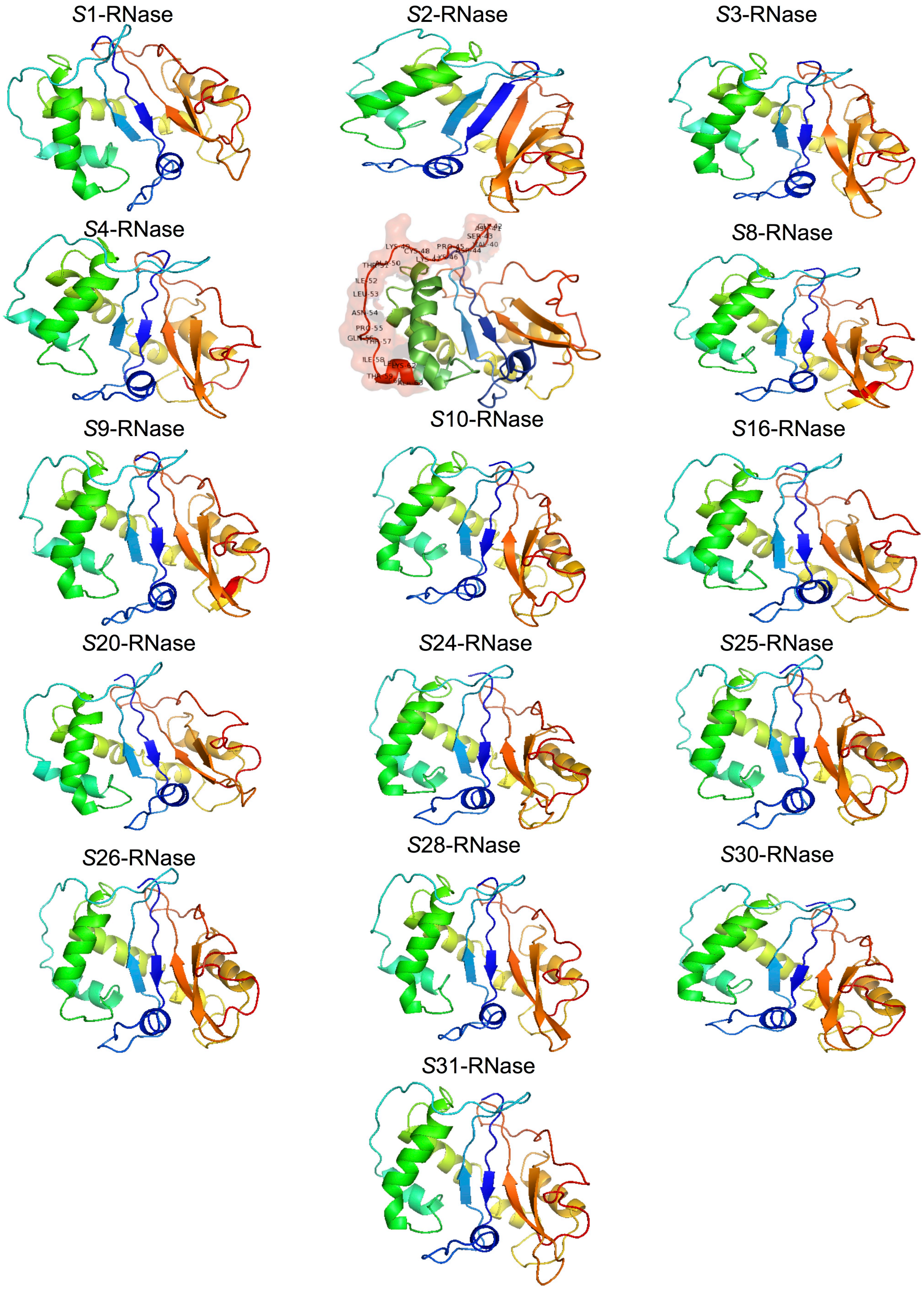

Supplementary Figure 4 | The predicted tertiary structures of *Malus* SRNases. The structures were generated by I-TASSER[1] and contain α-helices and β-sheets. The final tertiary structures of the *Malus* SRNases predicted models contain 6-7 α-helices and 4-7 β-sheets. The folding topologies of the modelled structures were very similar to the topologies of *Momordica charantia* ribonuclease MC and MC1 based on the Dali server database (Supplementary Table 1). The location of HV region is shown as surface representation on the S10-RNase structure in red while the amino acid composition of HV is also marked. The structures were visualization in PyMol v1.4.1[18].

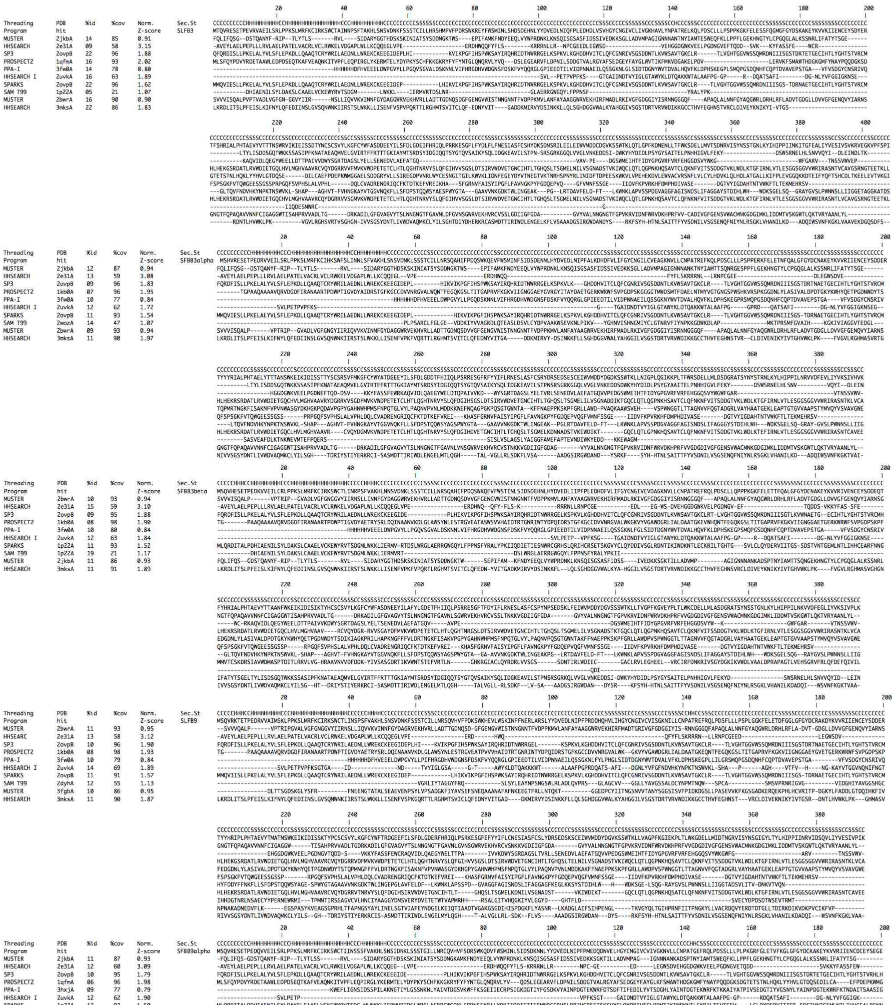

Supplementary Figure 5 | Amino acids threading alignment of the top-ten templates identified by LOMETS threading programs for modeling SLF/SFB/SFBBs tertiary structures. 2e31A: *Mus musculus* Skp1-Fbs1 complex[19], 2ovpB: *Homo sapience* Fbw7-Skp1-cyclin E complex[20], 1qfmA: *Sus scrofa* Prolyl oligopeptidase[21], 2wozA: *Rattus norvegicus* BTB-Kelch protein Krp1[22], 2uvkA: *Escherichia coli* beta-propeller protein YjhT[23], 3mksA: *Saccharomyces cerevisiae* Cdc4/Skp1-SCF-I2[24], 1kb0A: *Comamonas testosterone* Quinohemoprotein alcohol dehydrogenase[25], 1p22A: Human β-TrCP1-Skp1-beta-catenin complex[26], 2dyhA: *Mus musculus* Kelch-like ECH-associated protein 1[27].

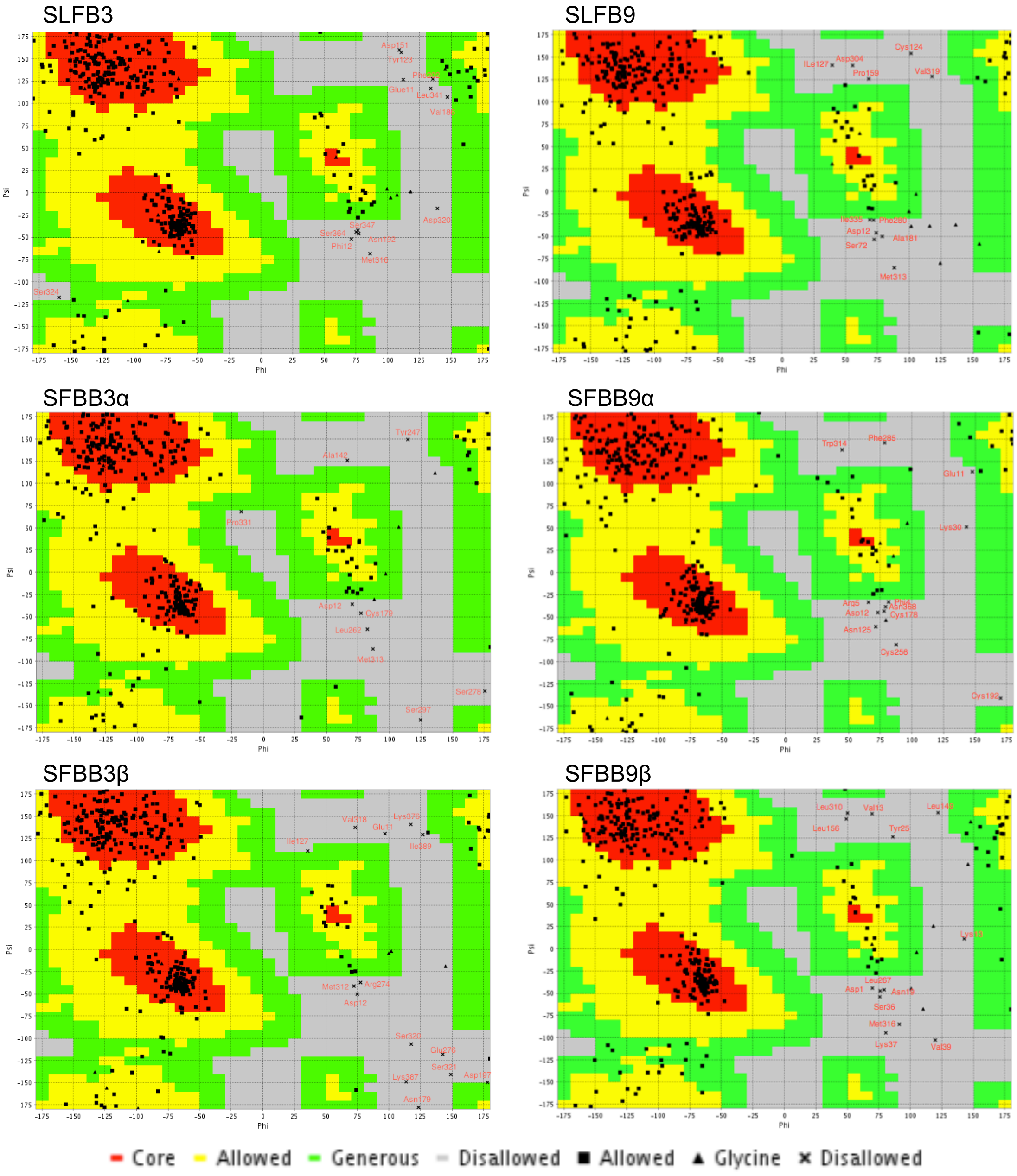

Supplementary Figure 6 | Ramachandran plots of the modeled SLF/SFBs. The red, yellow, green, and gray areas refer to core, allowed, general, and disallowed regions of the plot respectively. The plot was generated using PROCHECK[2] in VADAR v1.8[3].

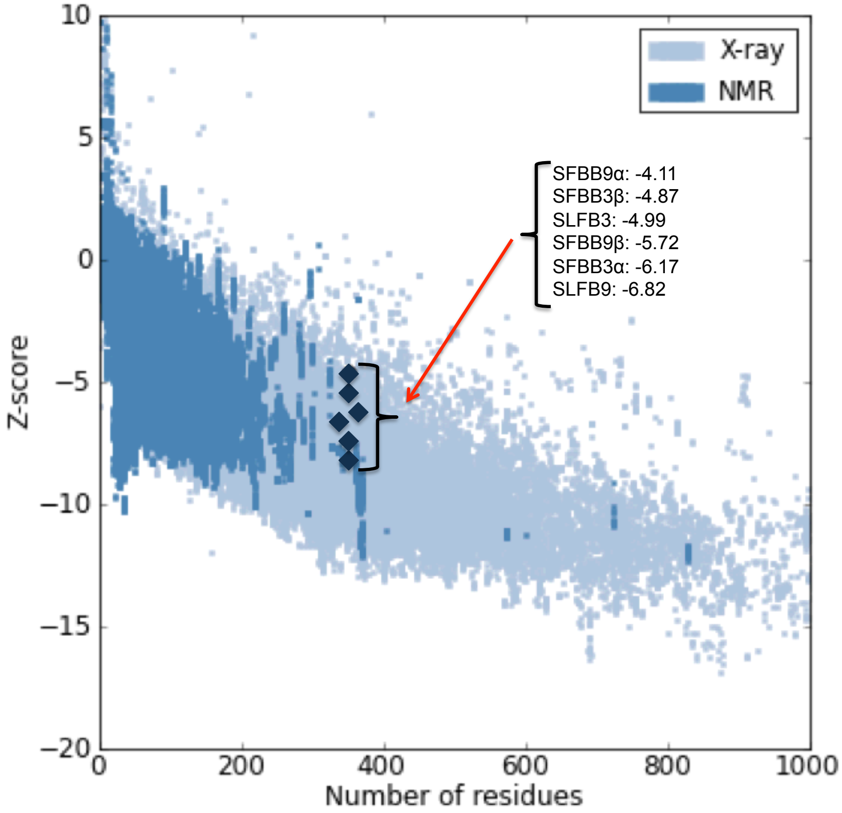

Supplementary Figure 7 | Z-score plot of all experimentally determined proteins. Protein structures from NMR experiments are coloured in dark blue whereas those from X-ray experiment are indicated by light blue. The location of SLF/SFBs z-score is within the boundaries of experimentally determined structures. The plot was generated using ProSa-web[4].

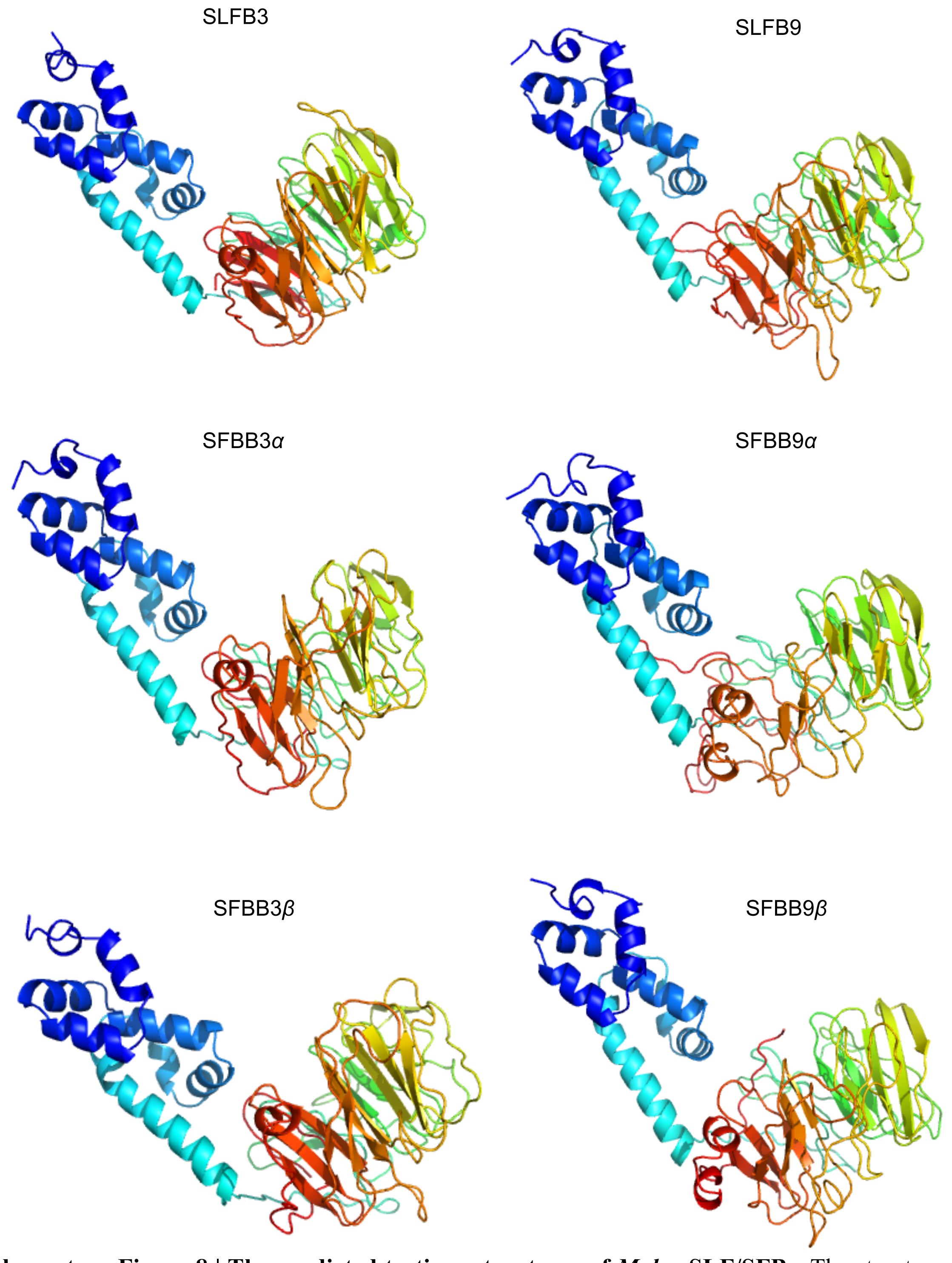

Supplementary Figure 8 | The predicted tertiary structures of *Malus* SLF/SFBs. The structures were generated by I-TASSER[1] and contain α-helices in the N-terminus and β-sheets in the C-terminus. The final tertiary structures of the *Malus* SLF/SFBs predicted models contain 8-10 α-helices and 17-23 β-sheets. The folding topologies of the modelled structures were very similar to the topologies of F-BOX/WD-repeat proteins based on the Dali server database (Supplementary Table 3). The structures were visualization in PyMol v1.4.1[18].

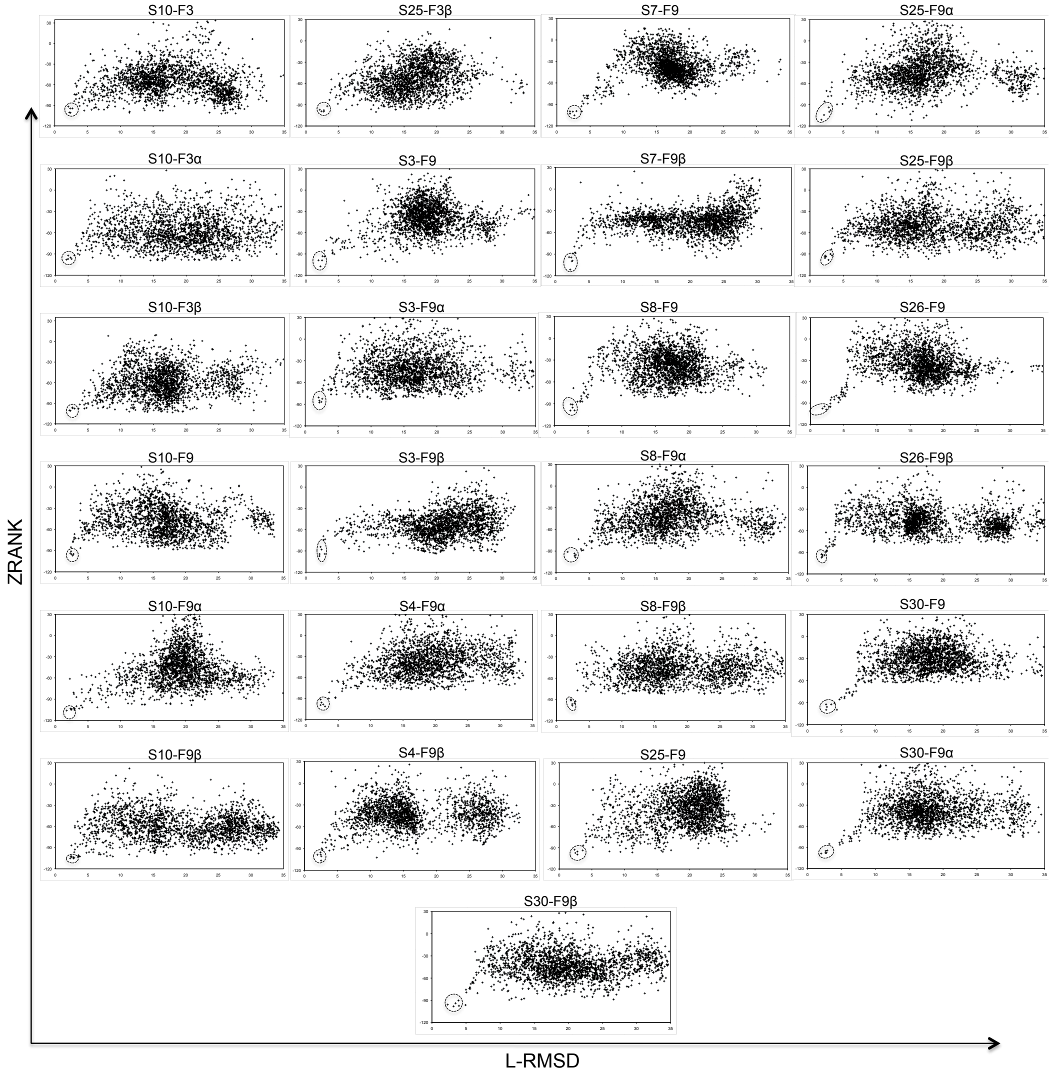

Supplementary Figure 9 | Scatter plot of the lowest energy scoring poses among the 2000 poses generated by ZDOCK. The top five models are indicated by a circle while ‘S’ and ‘F’ refer to SRNase and SLF/SFB, respectively.

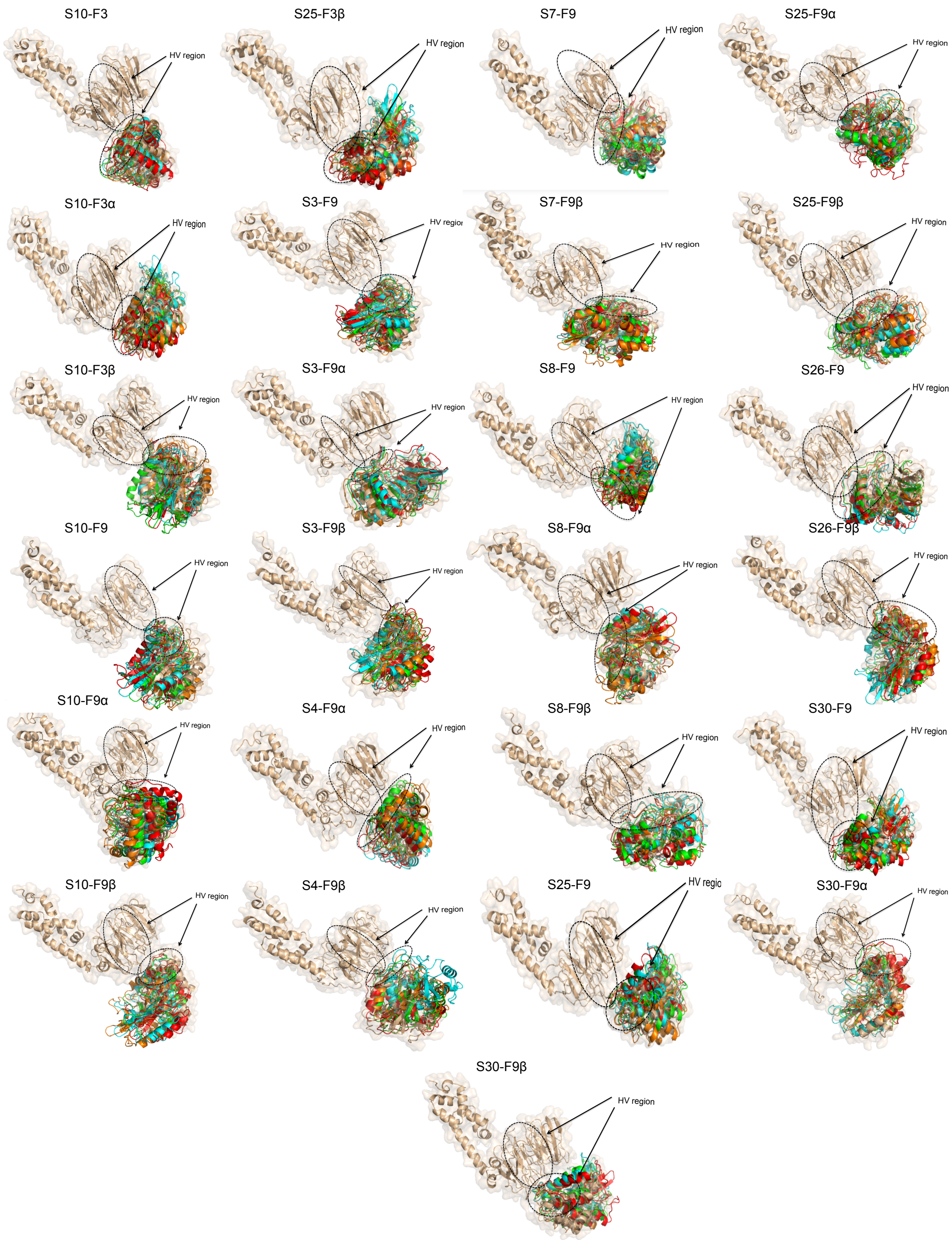

Supplementary Figure 10 | Three-dimensional illustration of SLF/SFB and SRNase complexes. 'S' and 'F' refer to SRNase and SLF/SFB, respectively. SLF/SFB structure and superimposed structure of the top five models of SRNase are located on the top and bottom of the complexes, respectively. The hypervariable regions (HVs) of SLF/SFB and SRNase structures are pointed using the circles. The position of circles against each other is showed the binding topology in the complex. The structures were visualization in PyMol v1.4.1[18].

Supplementary Tables:

Supplementary Table 1 | GenBank accession numbers of *Malus* SRNases and SLF/SFBs used in this study.

S-allele	Accession No.	S-allele	Accession No.
S1	D50837	S25	AB428431
S2	U12199	S26	AF016918
S3	U12200	S28	AF1748
S4	AF327223	S30	AB035928
S7	U19792	S31	DQ135990
S8	AY744080	SLFB3	GU345811
S9	U19793	SLFB9	AB270792
S10	AF239809	SFBB3α	AB270795
S16	AB428429	SFBB9α	AB270793
S20	AB019184	SFBB3β	AB270796
S24	AF016920	SFBB9β	AB270794

Supplementary Table 2 | Top templates identified by LOMETS to model the tertiary structures of SRNases.

Protein Hit	PDB	%Identity	%Coverage	Norm. Z-score
S1-RNase	1iqqA, 1iybA, 1bk7A, 1ucdA	30-62	93-99	4.20-5.32
S2-RNase	1iqqA, 1iybA, 1bk7A	27-63	93-100	4.09-5.38
S3-RNase	1iqqA, 1iybA, 1bk7A, 1ucdA	31-63	93-100	4.16-5.47
S4-RNase	1iqqA, 1iybA, 1bk7A, 1vczA	28-72	93-100	4.04-5.50
S7-RNase	1iqqA, 1iybA, 1bk7A, 1ucdA	28-62	93-100	4.08-5.38
S8-RNase	1iqqA, 1iybA, 1bk7A, 1ucdA	27-58	93-100	4.09-5.43
S9-RNase	1iqqA, 1iybA, 1bk7A	27-64	93-100	4.19-5.41
S10-RNase	1iqqA, 1iybA, 1bk7A, 1ucdA	31-64	93-100	4.11-5.45
S16-RNase	1iqqA, 1iybA, 1bk7A, 1iooA	26-72	93-100	4.00-5.51
S20-RNase	1iqqA, 1iybA, 1bk7A, 1ucdA	30-61	93-99	4.17-5.26
S24-RNase	1iqqA, 1iybA, 1bk7A, 1ucdA	28-60	93-99	4.20-5.30
S25-RNase	1iqqA, 1iybA, 1bk7A	30-61	95-100	4.13-5.41
S26-RNase	1iqqA, 1iybA, 1bk7A	27-66	95-99	4.29-5.31
S28-RNase	1iqqA, 1iybA, 1bk7A	30-63	94-99	4.28-5.10
S30-RNase	1iqqA, 1iybA, 1bk7A, 1iooA	24-95	93-100	4.05-5.63
S31-RNase	1iqqA, 1iybA, 1bk7A, 1ucdA	30-61	93-99	4.20-5.25

Supplementary Table 3 | Model evaluation scores for the predicted SRNases tertiary structures using I-TASSER.

*S*RNase	C-score	TM-score	RMSD (Å)	Folding energy	Core Ram.	Prosa-web Z-score
*S*1	1.79	0.97±0.05	1.9±1.6	-175.30	98	-6.59
*S*2	1.81	0.97±0.05	1.9±1.6	-180.09	97	-7.16
*S*3	1.82	0.97±0.05	1.9±1.6	-178.45	96	-6.59
*S*4	1.84	0.97±0.05	1.8±1.5	-166.45	98	-6.43
*S*7	1.81	0.97±0.05	1.9±1.6	-171.04	96	-6.43
*S*8	1.82	0.97±0.05	1.9±1.6	-171.52	97	-6.92
*S*9	1.82	0.97±0.05	1.9±1.6	-181.15	97	-7.09
*S*10	1.82	0.97±0.05	1.9±1.6	-175.73	96	-6.89
*S*16	1.84	0.97±0.05	1.8±1.5	-174.39	98	-6.69
*S*20	1.79	0.97±0.05	1.9±1.6	-174.37	98	-6.43
*S*24	1.78	0.97±0.05	1.9±1.6	-168.89	97	-6.46
*S*25	1.82	0.97±0.05	1.9±1.6	-168.15	97	-6.65
*S*26	1.81	0.97±0.05	1.9±1.6	-173.93	98	-6.49
*S*28	1.76	0.96±0.05	2.0±1.6	-175.80	97	-7.08
*S*30	1.86	0.98±0.05	1.8±1.5	-172.51	98	-6.11
*S*31	1.78	0.97±0.05	1.9±1.6	-176.70	98	-6.42

Supplementary Table 4 | Proteins with similar folding topologies to the predicted tertiary structures of SRNases.

Protein	PDB	Z-score	RMSD (Å)	Alig. L.	No. Res.	% Id.	Description
*S*1-RNase	1ucgB, 1j1fA, 1j1gA, 1v9hA, 1uccA	22.7-22.9	2.1-2.3	185-186	190-193	29-30	Ribonuclease MC and MC1
*S*2-RNase	1ucgB, 1j1fA, 1j1gA, 1v9hA, 1uccA	22.7-22.9	2.1-2.2	184-185	190-193	30-31	Ribonuclease MC and MC1
*S*3-RNase	1ucgB, 1j1fA, 1j1gA, 1v9hA, 1dixA	22.9-23.1	2.1-2.3	185-189	190-208	28-31	Ribonuclease MC, MC1 and LE
*S*4-RNase	1ucgB, 1j1fA, 1j1gA, 1v9hA, 1uccA	22.8-23.0	2.1 -2.2	184	190-193	29	Ribonuclease MC and MC1
*S*7-RNase	1ucgB, 1j1fA, 1j1gA, 1v9hA, 1uccA	23.3-23.5	2.0-2.2	184-185	190-193	30-31	Ribonuclease MC and MC1
*S*8-RNase	1ucgB, 1j1fA, 1j1gA, 1uccA, 1dixA	23.0-23.1	2.1-2.2	185-190	190-208	26-32	Ribonuclease MC, MC1 and LE
*S*9-RNase	1ucgB, 1j1fA, 1j1gA, 1v9hA, 1uccA	23.0-23.2	2.0-2.2	184-185	190-193	29	Ribonuclease MC and MC1
*S*10-RNase	1ucgB, 1j1fA, 1j1gA, 1uccA, 1bk7A	22.5-22.6	2.1-2.2	184-185	190-191	29-30	Ribonuclease MC, MC1 and LE
*S*16-RNase	1ucgB, 1j1fA, 1j1gA, 1v9hA, 1uccA	23.0-23.2	2.1-2.3	183-184	190-193	30-31	Ribonuclease MC and MC1
*S*20-RNase	1ucgB, 1j1fA, 1j1gA, 1v9hA, 1uccA	23.0-23.2	2.1-2.3	185-186	190-193	28-29	Ribonuclease MC and MC1
*S*24-RNase	1ucgB, 1j1fA, 1j1gA, 1v9hA, 1uccA	23.2-23.4	2.1-2.2	185-186	190-193	28-29	Ribonuclease MC and MC1
*S*25-RNase	1ucgB, 1j1fA, 1j1gA, 1dixA, 1iybA	22.7-22.8	2.1-2.2	185-190	190-208	28-31	Ribonuclease MC, MC1 and LE
*S*26-RNase	1ucgB, 1j1fA, 1j1gA, 1v9hA, 1uccA	22.9-23.1	2.0-2.2	184-185	190-193	30	Ribonuclease MC and MC1
*S*28-RNase	1ucgB, 1j1fA, 1j1gA, 1v9hA, 1uccA	23.0-23.2	2.1-2.3	185 -186	190-193	29-30	Ribonuclease MC and MC1
*S*30-RNase	1ucgB, 1j1fA, 1j1gA, 1v9hA, 1uccA	22.7-22.9	2.1-2.3	184	190-193	30-31	Ribonuclease MC and MC1
*S*31-RNase	1ucgB, 1j1fA, 1j1gA, 1v9hA, 1uccA	23.2-23.4	2.1-2.3	185 -186	190-193	28-29	Ribonuclease MC and MC1

Supplementary Table 5 | Best templates identified by LOMETS to model the tertiary structures of SLF/SFBs.

Protein	PDB	% Identity	% Coverage	Norm Z-score
*S*LFB3	2e31A, 2ovpB, 2uvkA, 3mksA,1qfmA,1p22A	8.0-20	21-96	1.07-3.15
*S*FBB3α	2e31A, 2ovpB, 2uvkA, 3mksA, 1kb0A, 2wozA	7.0-22	47-96	1.07-3.08
*S*FBB3β	2e31A, 2ovpB, 2uvkA, 3mksA, 1p22A	6.0-21	21-98	1.17-3.10
*S*LFB9	2e31A, 2ovpB, 2uvkA, 3mksA, 1kb0A, 2dyhA	8.0-14	55-96	1.13-3.12
*S*FBB9α	2e31A, 2ovpB, 2uvkA, 3mksA, 1qfmA, 2wozA, 1p22A	6.0-13	46-96	1.06-3.09
*S*FBB9β	2e31A, 2ovpB, 2uvkA, 3mksA, 1kb0A, 2wozA	7.0-14	46-97	1.20-3.07

Supplementary Table 6 | Quality assessment of the top-five predicted models of SLF/SFBs. The scores were generated using I-TASSER suite and the QMEANnorm score was calculated using QMEAN server.

Protein Model	C-score	Folding Energy	QMEANnorm Score
**SLFB3**			
model1	-2.26	-396.80	0.24
model2	-2.26	-365.14	0.19
model3	-2.26	-378.97	0.20
model4	-2.26	-338.55	0.34
model5	-3.97	-349.09	0.26
**SFBB3α**			
model1	-1.34	-352.00	0.30
model2	-1.34	-351.73	0.26
model3	-1.34	-325.71	0.22
model4	-1.65	-359.94	0.28
model5	-2.95	-348.25	0.24
**SFBB3β**			
model1	-1.61	-379.31	0.25
model2	-1.61	-351.59	0.23
model3	-1.61	-365.67	0.21
model4	-1.61	-338.80	0.21
model5	-3.23	-357.62	0.24
**SLFB9**			
model1	-2.58	-369.04	0.17
model2	-2.58	-372.79	0.26
model3	-2.58	-372.49	0.25
model4	-2.58	-342.56	0.19
model5	-4.01	-356.69	0.33
**SFBB9α**			
model1	-2.68	-358.92	0.24
model2	-2.68	-358.23	0.19
model3	-2.68	-362.86	0.17
model4	-2.68	-309.98	0.28
model5	-4.18	-357.60	0.35
**SFBB9β**			
model1	-2.61	-376.35	0.20
model2	-2.61	-353.26	0.25
model3	-2.61	-363.12	0.23
model4	-2.61	-357.24	0.24
model5	-4.29	-355.06	0.24

Note: The best model among the top-five predicted models for each SLF/SFB is shaded.

Supplementary Table 7 | Proteins with similar folding topologies to the predicted structures of SLF/SFBs.

Protein	PDB	Z-score	RMSD (Å)	Alig. L.	No. Res.	% Id.	Description
*S*LFB3	1p22A, 1vyhD, 3ow8A, 3dm0A, 2hesX	32.0-50.9	1.2-8.0	270-382	293-675	7.0-12	F-BOX/WD-repeat protein 1A, Platelet-activating factor acetylhydrolase IB beta subunit, WD repeat-containing protein 61, YDR267CP, Maltos-binding periplasmic protein fused with RACK1
*S*FBB3α	1p22A, 1vyhD, 1vyhS, 1vyhT, 1vyhC	28.2-46.0	1.4-2.1	261-370	310-402	8.0-11	F-BOX/WD-repeat protein 1A, Platelet-activating factor acetylhydrolase IB β subunit
*S*FBB3β	1p22A, 1vyhD, 1vyhS, 1vyhT, 3ow8A	25.7-41.6	1.7-2.4	256-368	293-402	7.0-10	F-BOX/WD-repeat protein 1A, Platelet-activating factor acetylhydrolase IB β subunit, WD repeat-containing protein 61
*S*LFB9	1p22A, 1vyhD, 1vyhC, 1vyhP, 1vyhH	29.1-45.7	1.3-2.2	265-365	310-402	4.0-11	F-BOX/WD-repeat protein 1A, Platelet-activating factor acetylhydrolase IB β subunit
*S*FBB9α	1p22A, 1vyhD, 1vyhC, 1vyhS, 2g9aA	26.6-44.5	1.2-2.0	251-362	310-402	7.0-10	F-BOX/WD-repeat protein 1A, Platelet-activating factor acetylhydrolase IB β subunit, WD-repeat protein 5
*S*FBB9β	1p22A, 1vyhG, 1vyhT, 3ow8A, 3u5gG	23.5-40.6	1.5-2.5	239-402	293-402	7.0-9.0	F-BOX/WD-repeat protein 1A, Platelet-activating factor acetylhydrolase IB β subunit, WD repeat-containing protein 61, 40S ribosomal protein S0-A

References

1 Roy, A., Kucukural, A. & Zhang, Y. I-TASSER: a unified platform for automated protein structure and function prediction. *Nat Protoc* **5**, 725-738, (2010).

2 Laskowski, R. A., MacArthur, M. W., Moss, D. S. & Thornton, J. M. PROCHECK: a program to check the stereochemical quality of protein structures. *J Appl Crystallogr* **26**, 283-291, (1993).

3 Willard, L. *et al.* VADAR: a web server for quantitative evaluation of protein structure quality. *Nucleic Acids Res* **31**, 3316-3319, (2003).

4 Wiederstein, M. & Sippl, M. J. ProSA-web: interactive web service for the recognition of errors in three-dimensional structures of proteins. *Nucleic Acids Res* **35**, W407-W410, (2007).

5 Zhang, C. & Zhang, F. The Multifunctions of WD40 Proteins in Genome Integrity and Cell Cycle Progression. *J Genomics* **3**, 40-50, (2015).

6 Ashkani, J. & Rees, D. J. G. A Comprehensive Study of Molecular Evolution at the Self-Incompatibility Locus of Rosaceae. *J Mol Evol* **82**, 128-145, (2016).

7 Mayrose, I., Mitchell, A. & Pupko, T. Site-Specific Evolutionary Rate Inference: Taking Phylogenetic Uncertainty into Account. *J Mol Evol* **60**, 345-353, (2005).

8 Vieira, F. G., Sanchez-Gracia, A. & Rozas, J. Comparative genomic analysis of the odorant-binding protein family in 12 Drosophila genomes: purifying selection and birth-and-death evolution. *Genome Biol* **8**, R235-R235, (2007).

9 Tao, R. & Iezzoni, A. F. The S-RNase-based gametophytic self-incompatibility system in Prunus exhibits distinct genetic and Molecular features. *Sci Hort* **124**, 423-433, (2010).

10 Wilcoxon, F. Individual comparisons by ranking methods. *Biometr Bull* **1**, 80-83, (1945).

11 R-Core-Team (2013). R: A language and environment for statistical computing. R Foundation for Statistical Computing. R Foundation for Statistical Computing, Vienna, Austria. URL http://www.R-project.org/

12 Matsuura, T. *et al.* Crystal structure at 1.5 Å resolution of Pyrus pyrifolia pistil ribonuclease responsible for gametophytic self-incompatibility. *J Biol Chem* **276**, 45261-45269, (2001).

13 Kawano, S., Kakuta, Y. & Kimura, M. Guanine Binding Site of the Nicotiana glutinosa Ribonuclease NW Revealed by X-Ray Crystallography. *Biochemistry* **41**, 15195-15202, (2002).

14 Nakagawa, A. *et al.* Crystal structure of a ribonuclease from the seeds of bitter gourd (Momordica charantia) at 1.75 Å resolution. *BBA-Protein Struct M* **1433**, 253-260, (1999).

15 Suzuki, A. *et al.* Crystal Structures of the Ribonuclease MC1 from Bitter Gourd Seeds, Complexed with 2′-UMP or 3′-UMP, Reveal Structural Basis for Uridine Specificity. *Biochem Biophys Res Commun* **275**, 572-576, (2000).

16 Kawano, S., Kakuta, Y., Nakashima, T. & Kimura, M. Crystal Structures of the Nicotiana glutinosa Ribonuclease NT in Complex with Nucleoside Monophosphates. *J Biochem* **140**, 375-381, (2006).

17 Ida, K. *et al.* Crystallization and preliminary X-ray crystallographic analysis of S-allelic glycoprotein S(F11)-RNase from Nicotiana alata. *Acta Crystallogr D Biol Crystallogr* **57**, 143-144, (2001).

18 Schrodinger, L. L. C. *The PyMOL Molecular Graphics System, Version~1.3r1* (2010).

19 Mizushima, T. *et al.* Structural basis for the selection of glycosylated substrates by SCFFbs1 ubiquitin ligase. *Proc Natl Acad Sci* **104**, 5777-5781, (2007).

20 Hao, B., Oehlmann, S., Sowa, M. E., Harper, J. W. & Pavletich, N. P. Structure of a Fbw7-Skp1-cyclin E complex: multisite-phosphorylated substrate recognition by SCF ubiquitin ligases. *Mol Biosyst cell* **26**, 131-143, (2007).

21 Fulop, V., Bocskei, Z. & Polgar, L. Prolyl oligopeptidase: an unusual beta-propeller domain regulates proteolysis. *Cell* **94**, 161-170, (1998).

22 Gray, C. H., McGarry, L. C., Spence, H. J., Riboldi-Tunnicliffe, A. & Ozanne, B. W. Novel β-Propeller of the BTB-Kelch Protein Krp1 Provides a Binding Site for Lasp-1 That Is Necessary for Pseudopodial Extension. *J Biol Chem* **284**, 30498-30507, (2009).

23 Severi, E. *et al.* Sialic Acid Mutarotation Is Catalyzed by the Escherichia coli. *J Biol Chem* **283**, 4841-4849, (2008).

24 Orlicky, S. *et al.* An allosteric inhibitor of substrate recognition by the SCFCdc4 ubiquitin ligase. *Nat Biotechnol* **28**, 733-737, (2010).

25 Oubrie, A., Rozeboom, H. J., Kalk, K. H., Huizinga, E. G. & Dijkstra, B. W. Crystal Structure of Quinohemoprotein Alcohol Dehydrogenase from Comamonas testosteroni : STRUCTURAL BASIS FOR SUBSTRATE OXIDATION AND ELECTRON TRANSFER. *J Biol Chem* **277**, 3727-3732, (2002).

26 Wu, G. *et al.* Structure of a beta-TrCP1-Skp1-beta-catenin complex: destruction motif binding and lysine specificity of the SCF (beta-TrCP1) ubiquitin ligases. *Mol Cell* **11**, 1445-1456, (2003).

27 Tong, K. I. *et al.* Different Electrostatic Potentials Define ETGE and DLG Motifs as Hinge and Latch in Oxidative Stress Response. *Mol Cell Biol* **27**, 7511-7521, (2007).
